# Editors' Note

**DOI:** 10.5195/ijt.2022.6533

**Published:** 2022-12-13

**Authors:** Ellen R. Cohn, Jana Cason

## Journal Overview

The International Journal of Telerehabilitation (IJT) is a biannual journal dedicated to advancing telerehabilitation by disseminating peer-reviewed information about current research and practices. IJT is indexed by PubMed and Scopus.

IJT is published via the open journal system (OJS) and sponsored by the University of Pittsburgh's Office of Scholarly Communication and Publishing at the University Library System. This institutional support enables IJT to be subscription free and require no author fees.

The current issue of the multi-disciplinary IJT features impactful research across rehabilitation disciplines. As context, COVID-19, a global pandemic, even with the availability of vaccines, is still dramatically affecting healthcare worldwide to greater or lesser degrees, as well as the global economy. Submissions to IJT continue to increase, ostensibly to document how telerehabilitation is meeting these challenges. We regret that we are unable to publish the high volume of articles that are being submitted. Instead, we strive to promptly suggest other placements for work that is not central to the mission of the journal (i.e., telerehabilitation) or relevant to the larger IJT audience.

## International Readership and Authors

IJT enjoys a diverse international audience. We surmise that this wide readership is testimony to the expanding global relevance of telemedicine, telehealth, and telerehabilitation.

In this Fall 2022 issue, we are pleased to publish work by authors from Australia, Brazil, Canada, Japan, Pakistan, Saudi Arabia, South Africa, Turkey, Ukraine, and United States.

## For Your Consideration – Hybrid Approaches

Across the disciplines that employ telerehabilitation, there is increasing discussion about the value of hybrid practice — a combination of virtual and in-person care. Research is needed to confirm the value of hybrid approaches. IJT welcomes such research.

IJT editor Cohn recently had the opportunity to serve as a presenter/panelist in the workshop “The use of telehealth for disability evaluation in medicine and allied health,” sponsored by the National Academies of Sciences, Engineering, Medicine for the purpose of providing perspectives to the United States Social Security Administration. In their concluding remarks, workshop chair Allen Heinemann observed that:

telehealth encapsulates healthcare delivery via a wide range of modalities, including synchronous and asynchronous video, phone, email, text, and hybrid interactions between care providers and patients and among teams of allied health professionals. (Proceedings of a Workshop, National Academies Press, Washington, DC, p. 55)

Since its inception in 2008, IJT has embraced this wide range of modalities. Furthermore, as a journal dedicated to telerehabilitation, IJT's scope is not limited to telehealth-based rehabilitation. IJT also publishes telehealth-based research conducted in educational settings. The wide scope of expertise required in school-based settings is illustrated in an open-source audit protocol developed by Lundblom et al. (2022) to document whether school-based telepractice is comparable to in-person or hybrid practice. The School-based Telepractice Assessment (STA), archived in D-Scholarship under a Creative Commons Attribution License, an institutional repository at the University of Pittsburgh, can be freely downloaded from: http://d-scholarship.pitt.edu/41773/. All or parts of the STA can therefore be freely adapted with attribution for clinical or research purposes.

